# Exenatide Effects on Cardiometabolic Risk and Weight in Adolescents with Morbid Obesity and Type 2 Diabetes Mellitus: Two Case Reports

**DOI:** 10.23937/2572-4010.1510004

**Published:** 2015-05-06

**Authors:** Marisa Censani, Vivian L Chin, Ilene Fennoy

**Affiliations:** Department of Pediatrics, Division of Pediatric Endocrinology, Columbia University Medical Center, USA

**Keywords:** Exenatide, Obesity, Adolescents, Type 2 diabetes

## Abstract

**Objective:**

Glucagon-like peptide-1 (GLP-1) receptor agonists improve glycemic control and cardiometabolic risk factors in adults with type 2 diabetes mellitus, but pediatric data is sparse.

**Research design and methods:**

This is the first report to describe the effects of GLP-1 receptor agonist Exenatide on metabolic risk and weight in adolescents with morbid obesity (BMI>35kg/m^2^) and type 2 diabetes in the first 6 months after treatment initiation. Two patients with morbid obesity who failed conventional therapy with insulin glargine and Metformin were treated with the addition of Exenatide. Metabolic parameters were obtained at 3 and 6 months post intervention.

**Results:**

Improvements in cardiometabolic risk factors, such as fasting glucose and triglyceride levels, as well as HbA1c, were reached at 6 months compared to baseline.

**Conclusions:**

Our cases highlight the therapeutic potential of exenatide for adolescent patients with morbid obesity and type 2 diabetes.

## Introduction

Glucagon-like peptide-1 (GLP-1) receptor agonist Exenatide is an incretin mimetic approved by the US Food and Drug Administration in 2005 as an adjunct to diet and exercise for glycemic control in adults with type 2 diabetes. Improvements in cardiometabolic risk factors and weight in type 2 diabetes patients using Exenatide are well described in adults; however, reports in adolescents are limited to non-diabetic youth with obesity [1,2]. Given that GLP-1 agonist liraglutide, a medication for type 2 diabetes, has recently been approved by the FDA as the first injectable drug approved for the treatment of obesity, it is important to evaluate the effects of available options in this class of injectable medications. We report the first two cases of Exenatide therapy in adolescents with morbid obesity and type 2 diabetes to document improvement in cardiometabolic parameters as well as glycemic control within 6 months of therapy initiation.

## Research Design and Methods

Anthropometric measures (weight, waist circumference (WC), body mass index (BMI)) and blood pressure (BP) were measured as previously reported [3] at baseline prior to Exenatide treatment, and at 3 and 6 months post intervention. Blood samples were obtained between the hours of 8:00 and 10:00 AM at initial preoperative bariatric surgery evaluation after an overnight fast. A 75 gram, 2-hour oral glucose tolerance test (OGTT) was performed with insulin and glucose samples obtained at 0, 30, 60, and 120 minutes. Glucose, lipids, HbA1c, liver function tests, and basic metabolic panel were performed at the laboratory of New York Presbyterian Hospital. Insulin’s were performed at Esoterix Laboratories, Calabasas, CA.

## Results

### Case 1

A 16 year old Hispanic female was diagnosed with type 2 diabetes 24 months after laparoscopic adjustable gastric banding (LAGB). She presented to the Center for Adolescent Bariatric Surgery (CABS) for evaluation of severe morbid obesity with BMI 86.2kg/m^2^ (weight 201.7kg, height 153cm) and underwent LAGB at age 14 years 3 months. Weight problems were present from early infancy. She had Blount’s disease requiring 5 surgical procedures to correct leg bowing and remained with limited mobility. Family history was notable for type 2 diabetes, hypertension, and stroke.

At initial preoperative visit, laboratory evaluation revealed metabolic syndrome with hypertriglyceridemia (170mg/dl), low high-density lipoprotein (HDL) 35mg/dl, impaired fasting glucose (IFG) 106mg/dl, and increased WC 188cm in addition to impaired glucose tolerance (140mg/dl) with HbA1c 6.5% and c-reactive protein (CRP) 46.2mg/dl. Following surgery, she lost 13.2kg over 6 weeks, but by 24 months post-operatively, her weight had increased to 17.4kg above her preoperative weight. A 2-hour OGTT demonstrated fasting glucose (FG) 188mg/dl, 2 hour glucose 293mg/dl and HbA1c 8.8% resulting in the diagnosis of type 2 diabetes. Metformin 850mg BID and Lantus 10 units were started, but she self discontinued Metformin due to gastrointestinal side effects.

Exenatide 5ug SQ twice daily (BID) was started as adjunctive therapy to Lantus 15 units at weight 209.0kg, and BMI 87.0kg/m^2^. After 6 months, she had a weight loss of 4.4kg, with improvements in BMI (−1.6kg/m^2^), FG (−56mg/dl), triglycerides (TG) (−68mg/dl), low-density lipoprotein (LDL) (−16mg/dl), total cholesterol (TC) (−31mg/dl), and CRP (−20%). She tolerated Exenatide and Lantus 15 units without complaint. She was lost to follow-up and returned one year later on Lantus and Metformin 1000mg twice daily with Exenatide self-discontinued.

With her weight 207.2kgs, HbA1c 11.2% and FG 297mg/dl, Exenatide 2mg once weekly (QW) was added to her Lantus and metformin regimen. At 3 months, her weight decreased to 198.9kgs (−8.3kgs) and FG decreased to 160mg/dl (−137). By 6 months, HbA1c substantially decreased to 8.4% with FG 103mg/dl and reached 7.6% by 9 months post therapy with stabilization of weight at 199.3kgs.

### Case 2

A 14 year old Hispanic female with a 3 year history of type 2 diabetes on once-daily DPP-4 inhibitor Sitagliptin presented for evaluation of morbid obesity with asthma, obstructive sleep apnea, reflux, and irregular menses. She was overweight since preschool age. Family history was notable for type 2 diabetes, obesity, hypertension, hyperlipidemia, and cholelithiasis.

At initial preoperative evaluation for bariatric surgery, weight was 129.3kg, height was 154.5cm, and BMI was 54.2kg/m^2^. Laboratory values were consistent with type 2 diabetes with HbA1c 10.7%, FG 210mg/dL, and 2 hour glucose 378mg/dL, and metabolic syndrome with TG 138mg/dL, HDL 39mg/dL, WC 142cm and elevated BP 120/83 (96^th^ percentile diastolic). Despite Sitagliptin100mg daily, Lantus 28 units daily and Metformin XR 1000mg, HbA1c remained between 9.9% and 10.3% for 6 months. She refused further increase in Metformin dose given gastrointestinal complaints. Sitagliptin was stopped and Exenatide 2mg QW was initiated as adjuvant treatment to Lantus 28 units and Metformin 1000mg daily at weight 131.1kg, BMI 54.4kg/m^2^, HbA1c 10.2%, and FG 163mg/dl. Within two months of treatment, HbA1c improved to 7.9%. After 6 months, she had sustained glycemic control with HbA1c 8.3% and notable improvements in cardiometabolic parameters including FG (−17mg/dl), insulin (−26uIU/ml), and TG (−12mg/dl). Patient’s weight was 131.5kg on day of gastric sleeve resection surgery with Exenatide QW discontinued following surgery due to normalization of blood glucose levels (HbA1c 5.8%) and weight 118.9kg noted at 2 months postoperatively.

## Conclusions

These are the first two cases in adolescents with morbid obesity and type 2 diabetes mellitus to document 6 month effects on cardiometabolic risk factors, weight, and glycemic control with GLP-1 receptor agonist Exenatide therapy. Improvements in metabolic parameters, HbA1c and weight have previously been documented in adult populations with type 2 diabetes. However, there is scarce longitudinal data pertaining to the pediatric population limited to two studies in non diabetic youth with obesity.

Exenatide is an incretin mimetic that shares approximately 50% sequence identity with human GLP-1, a gut derived factor rapidly secreted from the L-cells of the lower gut following meal ingestion and one of the most potent insulin secreting substances known [4–7]. GLP-1 secretion is demonstrated to be deficient in type 2 diabetes patients, but cellular responsiveness is not diminished (8). Adult longitudinal studies have documented normalized blood glucose, improved HbA1c, and weight loss in patients with type 2 diabetes [7–9]. Exenatide BID therapy has shown significantly reduced HbA1c [10,11] and improvements in TG (−39mg/dL), diastolicBP (−2.7mmHg), CRP (−44%) and HDL (+46mg/dL) [12]. In the (DURATION-1) study, [13] adult patients who completed 2 years of Exenatide QW treatment sustained long term improvements in HbA1c (−1.71 ± 0.08%), FG (−40.1 ± 2.9mg/dL), weight (−2.61 ± 0.52kg), systolic BP (−3.0 ± 1.0mmHg), and lipids, including TG (−15 ± 2.7%), TC (−8.6 ± 2.8mg/dL), and LDL (−4.5 ± 2.2mg/dL) compared with baseline [14].

Two pediatric studies have evaluated Exenatide as a weight-loss therapy in extreme pediatric obesity. A 6 month pilot study of 12 children and adolescents ages 9–16 years old with BMI ≥ 35kg/m^2^ analyzed the effects of Exenatide BID on BMI and cardiometabolic risk factors in nondiabetic youth. Compared to controls, Exenatide significantly improved BMI (−1.7kg/m^2^, p=0.01), weight (−3.9kg, p=0.02), fasting insulin (−7.5mU/l, p=0.02) and OGTT-derived insulin sensitivity (p=0.02) [1].

In a randomized, clinical trial by the same group, 26 adolescents 12–19 years old with severe obesity without diabetes were randomized to either three months of Exenatide or placebo then three months of open-label extension with Exenatide for all. Compliance was excellent and medication was well tolerated with mild to moderate events such as nausea (62% in Exenatide group vs. 31% in placebo group), abdominal pain (15% vs 23%), diarrhea (8% vs 31%), headache (23% vs 46%), and vomiting (31% vs 8%) with no episodes of hypoglycemia or pancreatitis. Compared to placebo, the Exenatide group had greater reduction in BMI (−1.13kg/m^2^, p=0.02), weight (−3.26kg, p=0.02) and final BMI of −4% after the 3 month open label extension with trend towards reduction in HbA1c (−0.11%, p=0.07) [2].

Our findings with Exenatide highlight the therapeutic potential of Exenatide and extend to the morbidly obese adolescent type 2 diabetes patient population previously described data regarding improvement in cardiometabolic risk factors in adults and in non-diabetic, obese adolescent patients. These findings suggest that Exenatide therapy may offer an additional treatment option that should be evaluated in larger, well-controlled trials of adolescent type 2 diabetes patients with morbid obesity.
